# Evolutionary Conserved Positions Define Protein Conformational Diversity

**DOI:** 10.1371/journal.pcbi.1004775

**Published:** 2016-03-23

**Authors:** Tadeo E. Saldaño, Alexander M. Monzon, Gustavo Parisi, Sebastian Fernandez-Alberti

**Affiliations:** Universidad Nacional de Quilmes, Bernal, Argentina; Koç University, TURKEY

## Abstract

Conformational diversity of the native state plays a central role in modulating protein function. The selection paradigm sustains that different ligands shift the conformational equilibrium through their binding to highest-affinity conformers. Intramolecular vibrational dynamics associated to each conformation should guarantee conformational transitions, which due to its importance, could possibly be associated with evolutionary conserved traits. Normal mode analysis, based on a coarse-grained model of the protein, can provide the required information to explore these features. Herein, we present a novel procedure to identify key positions sustaining the conformational diversity associated to ligand binding. The method is applied to an adequate refined dataset of 188 paired protein structures in their bound and unbound forms. Firstly, normal modes most involved in the conformational change are selected according to their corresponding overlap with structural distortions introduced by ligand binding. The subspace defined by these modes is used to analyze the effect of simulated point mutations on preserving the conformational diversity of the protein. We find a negative correlation between the effects of mutations on these normal mode subspaces associated to ligand-binding and position-specific evolutionary conservations obtained from multiple sequence-structure alignments. Positions whose mutations are found to alter the most these subspaces are defined as key positions, that is, dynamically important residues that mediate the ligand-binding conformational change. These positions are shown to be evolutionary conserved, mostly buried aliphatic residues localized in regular structural regions of the protein like β-sheets and α-helix.

## Author Summary

Conformational diversity of proteins can be associated to different aspects related to biological function. In particular, ligand binding can be analyzed in terms of the so-called ligand-free and ligand-bound conformations of a protein. These conformations co-exist as local minima within the energy landscape of proteins. The conformational change between them is achieved by their intramolecular vibrational dynamics. Therefore, it is expected that vibrational motions involved in the unbound-to-bound conformational change are evolutionary preserved. Herein, we present a novel procedure to identify key positions whose mutations have a significant effect on these particular vibrational motions. These key positions represent dynamically important sites that mediate the ligand-binding conformational change. They are shown to be evolutionary conserved, mostly buried aliphatic residues localized in regular structural regions of the protein like β-sheets and α-helix. These results contribute to improve our understanding on structure-function relationship as well as functional diversification during evolution.

## Introduction

Critical sites for protein function can be identified by sequence and structural alignment methods[1–2]. According to the neutral theory of molecular evolution[3], residues more relevant for function vary more slowly than less important ones. Nevertheless, these methods do not provide a complete information concerning the nature of the sequence-structure-function relationship and additional information related to proteins dynamics is required[4–12].

According to the generalized conformational selection model, the native state of proteins is represented by an ensemble of conformers in dynamics equilibrium[13–14]. In this model, ligands interacting with the proteins select the best conformer in terms of affinity, shifting the conformational equilibrium. Proteins are inherently dynamic entities and exist not as single structures, but as non-uniform distributions of multiple conformer populations. The protein dynamism plays an intricate role in defining the structure, function and evolution of individual proteins[15]. Therefore, the identification of special protein regions governing conformational changes results a major challenge.

Conformational diversity of proteins has been associated to different aspects related to biological function. Enzyme catalysis[16], signal transduction[17], protein recognition specificity[18], promiscuity[19], allosterism[20,21], origin of new protein functional adaptation and evolution[15,22,23] can be counted among others. In particular, ligand binding can be analyzed in terms of structural changes between the so-called ligand-free and ligand-bound conformations of a protein[24,25]. These conformers are characterized by their relative ligand affinities and their existences are extensively supported by a large variety of experimental evidence obtained from X-ray and cryo-electron microscope images, kinetic studies, single molecule fluorescence and NMR[26–29].

The need for considering different conformations in order to explain biological function could be generalized to most proteins. Computational tools for molecular docking[30], protein-protein interaction prediction[31], evaluation of protein structural models[32], prediction of observed substitution patterns of sequence divergence during evolution[33], and coevolutionary measurements between residues[22] are among the bioinformatic applications that address conformational diversity in order to improve their performance. More recently, a database of conformational diversity in the native state of proteins (CoDNaS)[34] with redundant collections of three-dimensional structures for the same proteins has been developed.

Ligand-free and ligand-bound conformations co-exist as local minima within the energy landscape of proteins[14]. The conformational change between them should be achieved by their intramolecular vibrational dynamics. The energy barriers that separate these conformers are commonly overcome by thermal fluctuations. The flexibility of the protein modulates the height of these barriers and the extent of the ensemble of conformations. Therefore, at least at the very beginning of the unbound-to-bound conformational change, the directions of their relative structural distortions should be dictated by dynamic fluctuations around the ligand-free conformation[35].

Normal mode analysis (NMA), based on a coarse-grained model of the protein, can provide the required information to explore the intrinsic dynamics within a folded protein[36–40]. The complex motions and fluctuations of proteins are decoupled into a linear combination of independent harmonic oscillators, i.e., the normal modes, each of them involving the concerted motions of many atoms. In that way, large-scale domain movements, involved in connecting the different conformational states related to function, can be identified[41–45]. A number of studies applied on vastly different enzymes show that conformational transitions are dominated by only a few low-frequency normal modes[35,46,47]. The effect of mutations on these collective and functionally relevant modes has been previously studied from different points of view. On one hand, the robustness of these modes to sequence variations has been reported[48–52]. Furthermore, normal mode conservation has been shown to increase linearly with collectivity, so that the slowest most collective modes are the most conserved ones[52]. Since these modes contribute the most in determining the overall flexibility B-factor profiles, the observed conservation of backbone flexibility can be explained [53,54]. On the other hand, the molecular understanding of the biological function requires identification of the network of residues that take part in function-related dynamics like substrate binding and product release, allosteric regulations, and folding. For example, residues that are dynamically important to ligand-binding have shown to be evolutionarily conserved[55]. By using the Structural Perturbation Method (SPM)[50,55,56], which proves the residue-specific response to perturbations, Zheng et al. were able to associate ligand-binding conformational changes to networks of functionally important residues[57].

The fact that normal modes provide a decoupled harmonic description of protein vibrations is fundamental to identify the individual equilibrium vibrational motions that participate of ligand-binding. Nevertheless, the identity of normal modes should be tracked after small perturbations and this is not a simple task since they can introduce rearrangements in their frequency ordering[51,58]. Besides, the complexity of the potential energy function of a protein may cause them to vary substantially and, eventually, to mix them strongly. In order to minimize these effects, in the present work we deal not with individual normal modes but with normal mode subspaces associated to ligand-binding. We present a procedure to define and compare normal mode subspaces associated to ligand-binding. Our definition of key positions, i.e. those that are dynamically important to ligand-binding, is based on the effect of mutations on these subspaces.

## Results and Discussion

### A. Identification of key positions in conformational transitions

**Fig 1:**
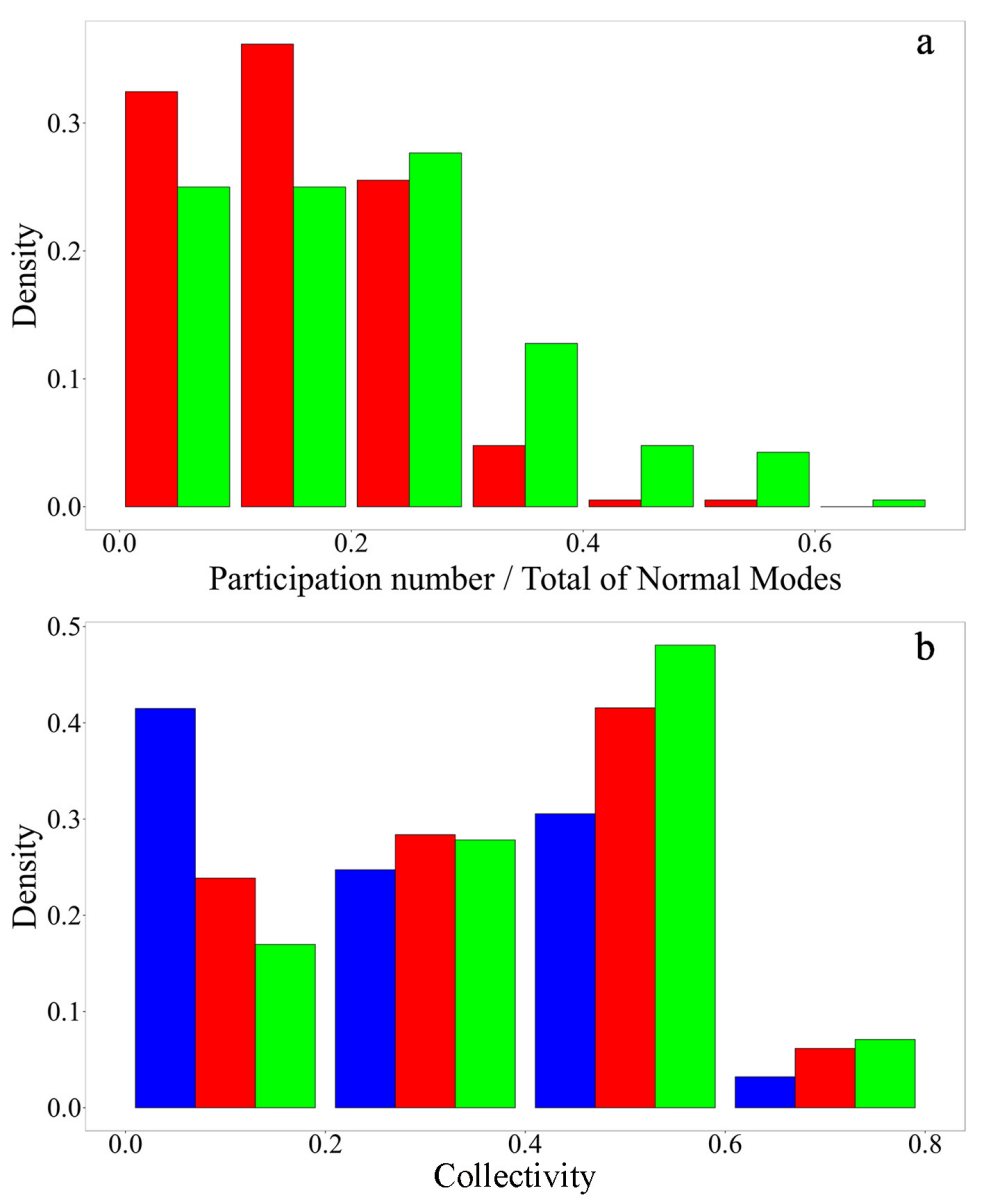
(a) Distribution of the fraction of normal modes involved in the conformational change calculated as values *P*_**q**_/3*N* (red), and the fraction of normal modes that participate significantly in the flexibility pattern calculated as *P*_**B**_/3*N* (green) obtained over all pairs of structures in our dataset. (b) Distribution of degree of collectivity, *κ*_*k*_, for each normal mode that participates in the conformational change (red), and each normal mode that significantly participates in the flexibility (B-factor) profile(green), and for all other modes (blue).

A number of previous studies have shown that ligand-associated conformational changes are dominated by only a few low-frequency normal modes[35,50,59,60,61]. Herein, the number of normal modes that span the subspace **S** associated to the conformational change is given by the value of the participation number *P*_**q**_ (see Methods). Fig 1(a) displays the distribution of the fraction of normal modes involved in the conformational change calculated as values *P*_**q**_/3*N* obtained over all pairs of structures in our dataset. Its average value is 0.15 ± 0.09, confirming the significant reduction of the corresponding original vibrational spaces. However, this is not always the case[46] as it is indicated by the tail at large values in our distribution, reaching the largest value of 0.59.

The composition of subspaces **S** is displayed in Fig 1(b) as the distribution of degree of collectivity, *κ*_*k*_, defined as[35]

$$\kappa_{k}=\frac{1}{N}\text{exp}\left(-\Sigma_{i=1}^{N}{\left(q_{i,k}^{r}\right)}^{2}\text{ln}{\left(q_{i,k}^{r}\right)}^{2}\right)$$ (1)

being ${\left(q_{i,k}^{r}\right)}^{2}\;=\;\;{\left(q_{i,k}^{x}\right)}^{2}+{\left(q_{i,k}^{y}\right)}^{2}+{\left(q_{i,k}^{z}\right)}^{2}$, and ${\left(q_{i,k}^{j}\right)}^{2}$ (*j* = *x*, *y*, *z*) are the components of the *i*th C_α_ residue in the *k* normal mode. Values of *κ*_*k*_ = *N*^−1^ corresponds to normal modes equally distributed throughout all the residues of the protein, and *κ*_*k*_ = 1 corresponds to normal modes involving the displacement of a single residue. In general, normal modes involved in the conformational change represent more collective vibrational motions than the rest of modes. The maximum of the distribution at 0.5 indicates that, on average, half of the residues participate in the concerted displacements described by each of these modes.

We have also explored the dependence of subspace **S** associated to ligand-binding with the global RMSD between conformers and protein size. In order to do that, we have considered both number and average degree of collectivity of modes that belong to subspace **S**. We have obtained negligible Spearman correlation coefficients of 0.03(p-value = 0.007) and -0.14 (p-value<2.2x10^-18^) for correlations of the collectivity of modes with global RMSD and protein size respectively. Furthermore, also a negligible correlation of 0.09(p-value = 0.23) has been obtained between participation number *P*_**q**_ and RMSD. Only a significant correlation of 0.49(p-value = 7.3x10^-13^) is obtained between *P*_**q**_ and protein size.

In order to differentiate normal modes involved in the conformational change from those that participate significantly in the flexibility pattern of each protein, vectors **B**^lf^ with elements ${\text{B}}_{\text{i}}^{\text{lf}}$ corresponding to the B-factors associated to each i^th^ residue have been expanded on the basis of ligand-free normal modes

$$B^{\text{lf}}=\Sigma_{k=1}^{3N-6}\left(B^{\text{lf}}\cdot q_{k}\right)q_{k}=\Sigma_{k=1}^{3N-6}\left(\Sigma_{j=1}^{3N}\left(B_{j}^{\text{lf}}q_{jk}\right)\right)q_{k}=\Sigma_{k=1}^{3N-6}b_{k}q_{k}$$ (2)

with

$$b_{k}=\Sigma_{j=1}^{3N}\left(B_{j}^{\text{lf}}q_{jk}\right)$$ (3)

In that way, the mode participation number *P*_**B**_ is defined as

$$P_{B}={\left(\Sigma_{k=1}^{3N-6}{\left(b_{k}\right)}^{4}\right)}^{-1}$$ (4)

with an equivalent interpretation as *P*_**q**_ described in Methods Section C. The first *P*_**B**_ modes ordered by index *f*_*k*_ in decreasing values of (*b*_*k*_)^2^ define the minimum subspace **S**_**B**_ of modes ${\left\{q_{f_{i}}\right\}}_{i=1,P_{B}}$ required to achieve a good description of the flexibility pattern. That is, **S**_**B**_ retains normal modes most involved in the B-factors of the ligand-free conformation.

Fig 1(a) shows the comparison between distributions of *P*_**q**_/3*N* and *P*_**B**_/3*N* values obtained over all pairs of structures in our dataset. As it is shown, larger subspaces of normal modes are required to achieve a good description of flexibility patterns than the ones associated to ligand-binding. Besides, Fig 1(b) shows the distribution of degree of collectivity for modes that belong to the subspace **S**_**B**_. The comparison with normal modes that participate in the conformational change indicates that modes involved in the flexibility pattern are only slightly less collective than those that participate in the flexibility patterns. This result is in good agreement with previous studies that shown that conformational changes are commonly associated to low-frequency normal modes[35,46]. Despite that, the participation of more localized normal modes during the conformational change is far from been negligible [46].

**Fig 2:**
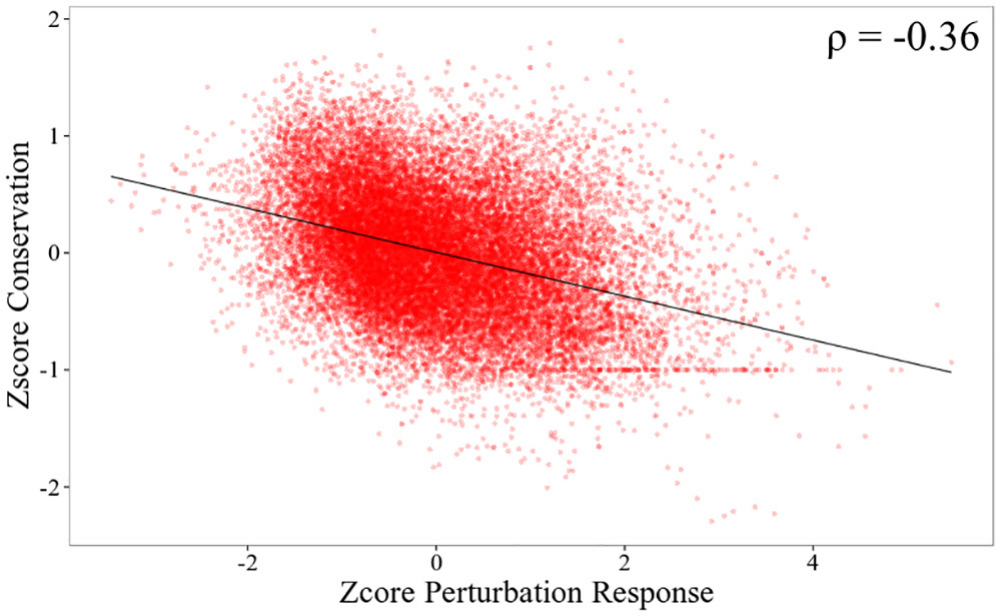
Effect of mutations on vibrations involved in ligand-binding ($Z_{\text{score}}^{S^{i}}$) vs. evolutionary conservation ($Z_{\text{score}}^{\text{evol},\text{i}}$). Linear regression line is included and linear correlation coefficient is shown in the top right corner.

As we mentioned before, conformational diversity of the native state plays a central role in modulating protein function. The co-existence of conformers with different ligand-affinities in a dynamical equilibrium is the basis for the conformational selection model for ligand binding. Internal protein motions associated to ligand-free conformation should guarantee unbound-to-bound conformational changes. Therefore, the effect of mutations on the subspace of normal modes **S** associated to ligand-binding should correlates with the evolutionary conservation of the corresponding sites. To investigate this, Fig 2 displays the relationship between effect of mutations on vibrations involved in ligand-binding ($Z_{\text{score}}^{S^{i}}$), and evolutionary conservation ($Z_{\text{score}}^{\text{evol},\text{i}}$). According to the larger collectivity reported for the normal modes that belong to the **S** subspace (see Fig 1(b)), and following previous studies of Zheng et al.[55], we average $Z_{\text{score}}^{S^{i}}$ and $Z_{\text{score}}^{\text{evol},\text{i}}$ over the neighbors of the *i*th residue within a radius of 7 Å. That is, we analyze spatial regions rather than individual residues. Furthermore, considering that mutations can lead to either stronger or weaker interactions between the *i*th residue and its spatial neighbors, our results correspond to the average obtained using a perturbation δγ±0.05. Our results do not significant change while using δγ within the range [±0.01: ±0.1]. In that way, we obtain a Spearman correlation coefficient ρ of -0.36 with a p-value 2.2x10^-16^. That is the stronger the impact that site-specific mutations have on the subspace of vibrations connected to ligand-binding, the more site-specific evolutionary conservation.

In order to analyze effects of protein size and global RMSD between conformers, we have analyzed the correlation between $Z_{\text{score}}^{S^{i}}$ and $Z_{\text{score}}^{\text{evol},\text{i}}$ for subsets of our protein dataset decomposed by pairs with (a) RMSD < RMSD_*max*_; (b) RMSD > RMSD_*max*_; (c) size < size_*max*_; (d) size > size_*max*_, being RMSD_*max*_ = 2.0Å and size_*max*_ = 80 the maximum of the distribution of the RMSD and size values obtained over all pairs of the final selected dataset. We obtained Spearman correlation coefficients of -0.32, -0.35, -0.30 and -0.34 for (a)-(d) subsets respectively. In all cases, a p-value <2.2x10^-16^ was obtained. Despite that our findings do not are not strongly influenced by neither the protein size nor the global RMSD between conformers, a slightly dependence is observed. That is, better correlations are observed for bigger proteins presenting larger structural distortions(RMSD) introduced by ligand binding.

Our findings allow us to identify key positions for the evolutionary conservation of the protein conformational diversity required for ligand binding. That is, positions whose mutations are found to alter the most the subspaces **S** containing the ligand-free normal modes involved in the unbound-to-bound conformational transition. For each pair of ligand-free and ligand-bound structures in our data set, we select the key positions as those ranked with the lowest 5% values of $Z_{\text{score}}^{S^{i}}$. Other choices for this cut off value between 1% and 10% do not qualitatively modify our results.

**Fig 3:**
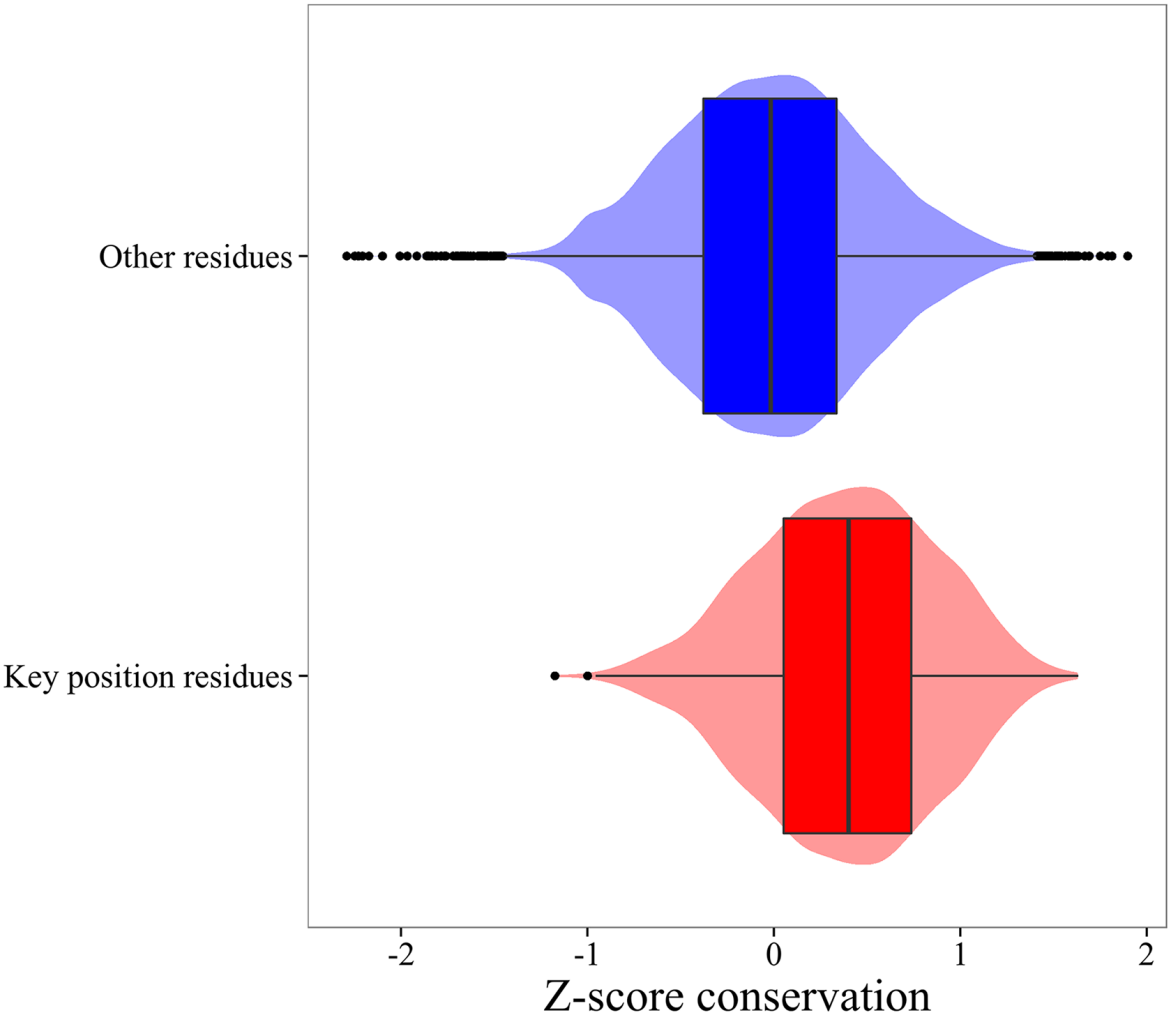
Distributions of the conservation measure $Z_{\text{score}}^{\text{evol},\text{i}}$ obtained for the selected key position residues (red), and all other residues (blue). The lower and upper "hinges" of the box correspond to the first and third quartile, and the black band inside the box is the median (Second quartile). The violin plot under the box plot shows the distribution of a given variable.

In Fig 3, we analyze the evolutionary conservation of these key residues relative to the rest of residues. The distribution of the values of $Z_{\text{score}}^{\text{evol},\text{i}}$ is significantly displaced toward larger values, indicating that key residues are evolutionary conserved. The difference between both distributions is statistically validated by the Kolmogorov-Smirnov statistic value of 0.31 with a p-value = 2.2x10^-16^.

At this point it is important to stress that the aim of the present work is not to fully explain the evolutionary conservation of position residues through their relevance on the protein conformational diversity. Previous works found that sequence evolutionary conservation results from multiple factors such as structural, dynamics, and/or functional features [62,63,64,65]. Our results displayed in Figs 2 and 3 emphasize that conformational diversity of the native state is just one of the many aspects that modulate protein function and, therefore, dynamically important residues or spatial regions associated to conformational diversity are more evolutionary constrained than other residues. Despite the existence of multiple sources of evolutionary conservation, it is noteworthy how the role on the conformational diversity of each residue position correlates with their evolutionary divergence. The *p*-values obtained in the analysis of Figs 2 and 3 quantify the statistical significance of our results, indicating that the observed data are inconsistent with the assumption that the null hypothesis is true.

### B. Characterization of detected key positions

**Fig 4:**
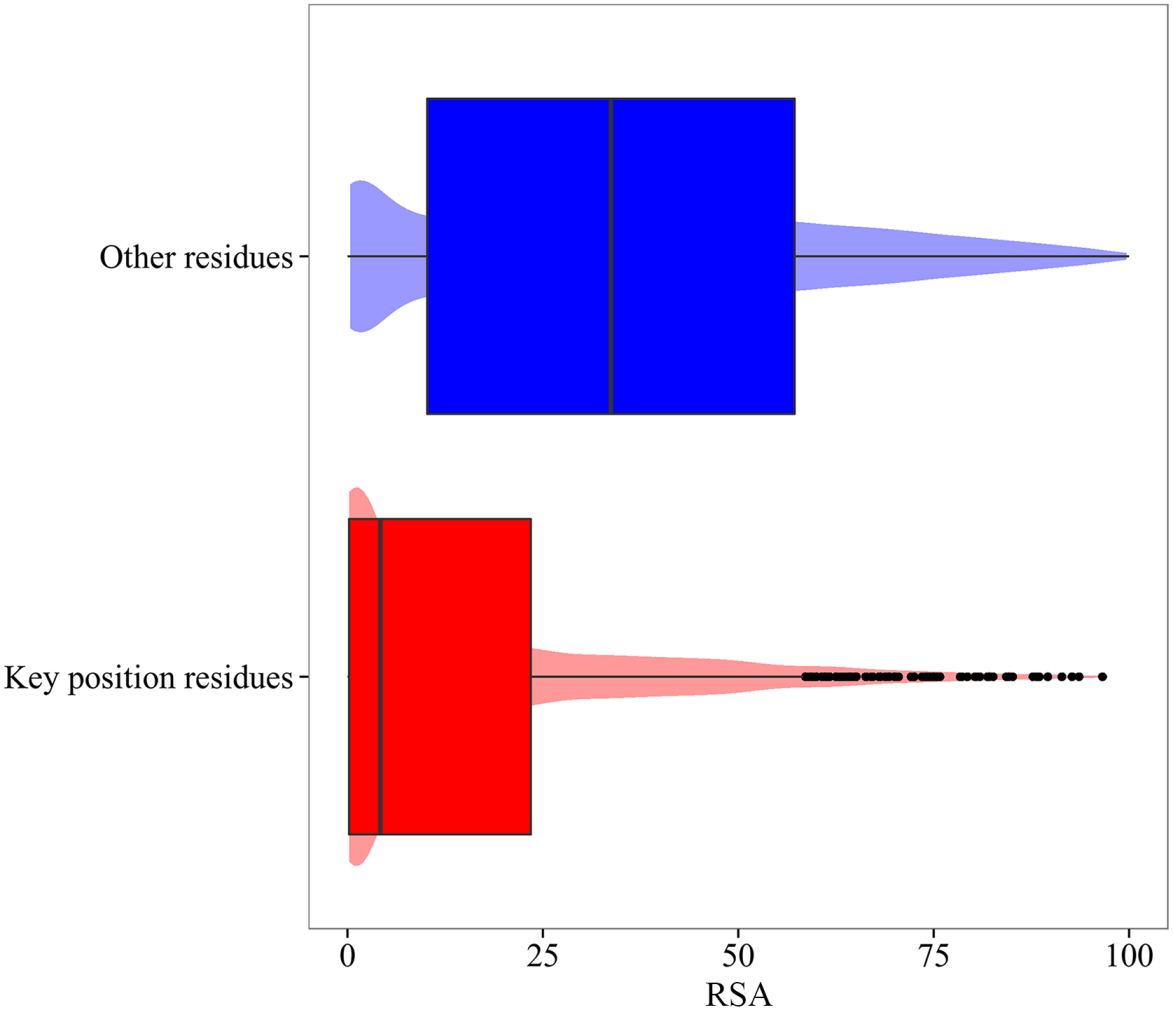
Relative accessible Surface Area (RSA) for key positions residues (red) and the rest of the positions in the protein (blue).

In what follows, we conduct different surveys to characterize the residues associated with key positions. Firstly, we analyze the incidence of the different amino acid types, defined as

$$I_{\propto }=\frac{\nu_{\alpha }^{\text{key}}}{\nu_{\alpha }}$$ (5)

where $\nu_{\alpha }^{\text{key}}$ is the frequency of the amino acid type α as a key position residue, and *v*_*α*_ the corresponding frequency in the rest of the residues. A value of *I*_*∝*_ > 1 indicates a higher frequency for the amino acid type α as a key position residue relative to its observed frequency in the protein dataset. Table 1 displays these values. Nonpolar amino acids Val, Ile, Leu, Met, Trp, and Phe are among the most frequently observed residues in the key positions detected, except Cys that presents the largest value of *I*_*∝*_ mainly due to its capacity for disulfide bond formation. This is in agreement with the comparison of the distribution of the Relative accessible Surface Area (RSA), calculated using the NACCESS program[66], for key position residues respect to the rest of residues in the protein (see Fig 4). Key positions are, in general, buried in the interior of the protein structure.

**Table 1:** Incidence of residues on key positions.

CYS	2.412	TYR	0.856
TRP	1.626	GLY	0.837
VAL	1.625	GLN	0.807
ILE	1.577	HIS	0.803
PHE	1.569	SER	0.792
LEU	1.432	ASP	0.706
MET	1.197	GLU	0.685
ASN	0.932	LYS	0.669
ALA	0.889	ARG	0.527
THR	0.883	PRO	0.438

At this point it is interesting to analyze the correlation among $Z_{\text{score}}^{S^{i}}$, $Z_{\text{score}}^{\text{evol},\text{i}}$, RSA and the number of inter-residue contacts for each residue of the dataset calculated using RING[67]. On one hand, the Pearson correlation coefficient between $Z_{\text{score}}^{S^{i}}$ and RSA results in a value of 0.48, while the corresponding value between $Z_{\text{score}}^{S^{i}}$ and the number of contacts per residue is -0.46. On the other hand, we obtain correlations of -0.27 between $Z_{\text{score}}^{\text{evol},\text{i}}$ and RSA, and 0.23 between $Z_{\text{score}}^{\text{evol},\text{i}}$ and the number of contacts per residue. That is, while either RSA and the number of contacts per residue strongly correlate with $Z_{\text{score}}^{S^{i}}$, both weakly correlate with $Z_{\text{score}}^{\text{evol},\text{i}}$. Considering our previous reported correlation of -0.36 between $Z_{\text{score}}^{S^{i}}$ and $Z_{\text{score}}^{\text{evol},\text{i}}$, we conclude that this value cannot be accounted by a simply evaluation of the RSA and number of contacts per residue. Besides, we also explore the relationship between either $Z_{\text{score}}^{S^{i}}$ and $Z_{\text{score}}^{\text{evol},\text{i}}$, and the RMSD^i^ per residue upon ligand binding. A strong correlation of 0.4 between $Z_{\text{score}}^{S^{i}}$ and the RMSD^i^ indicates that mutations on positions with little movement between the ligand-free and ligand-bound conformations will probably have a strong impact on vibrations associated to the conformational change. Nevertheless, a very weak correlation of -0.16 is obtained between $Z_{\text{score}}^{\text{evol},\text{i}}$ and RMSD^i^. That is, not all residues that barely move during the conformational change will be evolutionary conserved.

**Fig 5:**
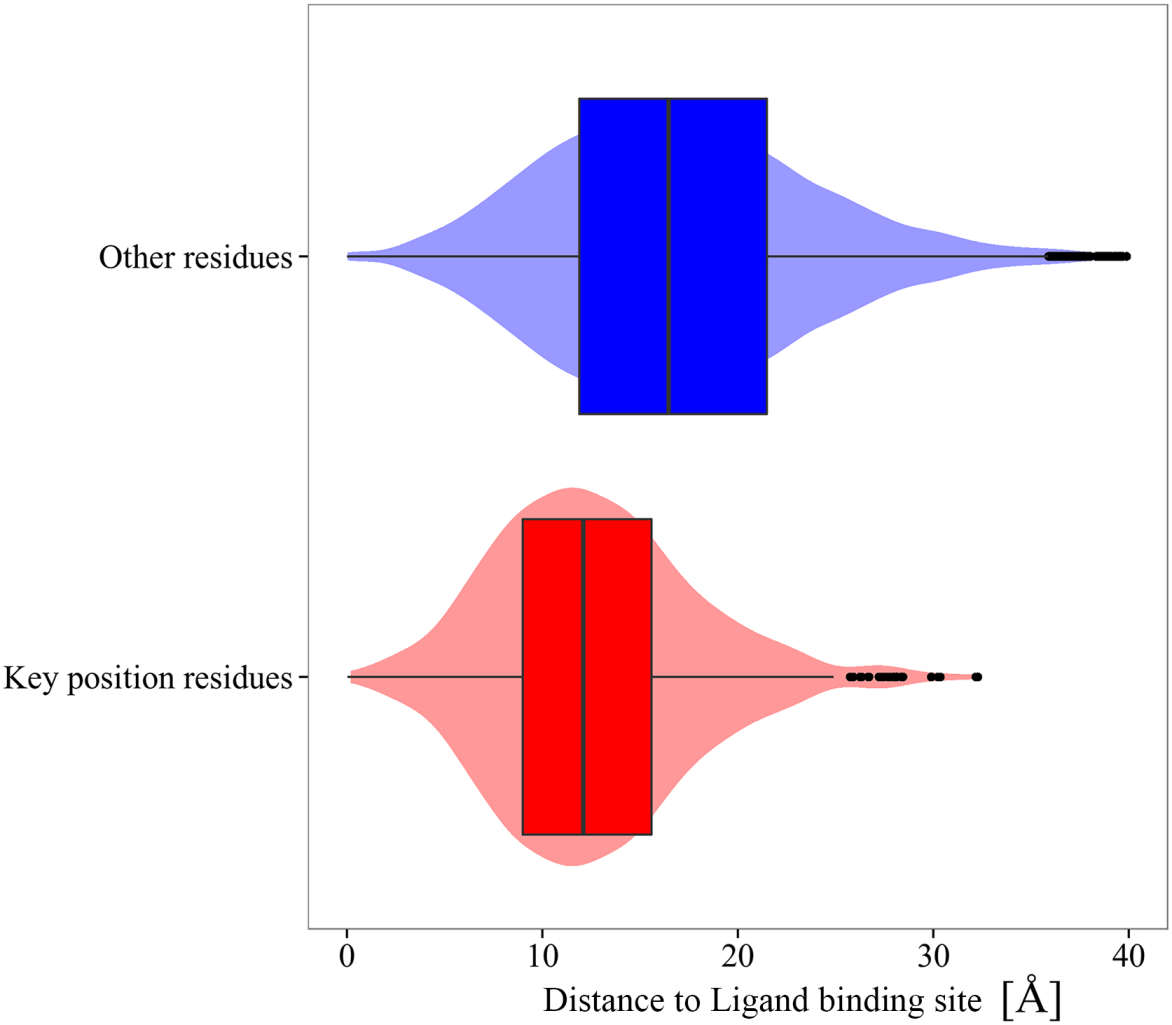
Distribution of the distances of key positions (red) and the rest of residues (blue) to the center of mass of protein active site.

BioLip dababase[68] has been used to obtained information concerning the active site of each protein in the dataset. Thus, the relative distances of key position to the center of mass of protein active site have been calculated. Fig 5 shows the distribution of these distances for both type of residues, that is, key position and the rest of residues in the protein. We observed that, in general, key position residues are closer to the active site without being part of it. Only ∼10% of the key position residues correspond to active site residues. The Pearson correlation coefficient between values of $Z_{\text{score}}^{S^{i}}$ and the distance to the center of mass of active sites is 0.39 with a p-value of 2.2x10^-16^. Previous studies have shown that active site residues are frequently related to residues that trigger conformational changes associated to ligand-binding [57,69,70,71]. Unbound-to-bound conformational transitions should introduce conformational changes in the active site leading to significant changes in the affinity for the ligand. Despite that, active-site residues only comprise a small fraction of the predicted key residues. This is in good agreement with previous results obtained by Zheng et al. [57]. Therefore, most of the evolutionary conserved key position residues are not directly associated to the enzyme catalysis.

Next, we analyze the association of key positions to the different secondary structure elements (SSE). For this purpouse, we use DSSP[72] (Dictionary of Protein Secondary Structure) that recognizes seven types of ordered local structure: H(α-helix), B(residue in isolated β-bridge), E(extended strand), G(3_10_ helix), I(π-helix), T(hydrogen bonded turn), S(bend), and N(unclassified). Table 2 shows the values of the incidence of key positions on the different SSEs, defined as

$$I_{SSE-\;X}=\frac{\nu_{SSE-X}^{\text{key}}}{\nu_{SSE-X}}$$ (6)

where $\nu_{SSE-X}^{\text{key}}$ is the frequency of key positions on the SSE-X, with X = H, B, E, G, I, T, or S, and *v*_SSE−x_ the corresponding frequency in the rest of the residues. A Value of *I*_SSE−x_ > 1 indicates a higher frequency for key positions to belong to that SSE relative to the observed frequency in the protein dataset. We observe that key positions are more frequently localized on extended strands (E), and also α-helices (H).

**Table 2:** Incidence of different residues on key positions related with SSEs.

E	2.087
H	1.146
B	1.019
G	0.608
N	0.547
S	0.355
T	0.349
I	0.000

Our measure of the structural distortions introduced by ligand-binding is given by the vector difference **v** whose elements are weighted by the corresponding B-factors as described in Methods. A scaling factor *w =* 0.01 is chosen as the value that maximize the correlation coefficient between $Z_{\text{score}}^{S^{i}}$ and $Z_{\text{score}}^{\text{evol},\text{i}}$. In this way, we avoid that our results can be skewed by any structural distortion not directly related to ligand binding. Loops and other flexible regions are inherently ruled out while domains and hinge regions are highlighted. Therefore, two kind of residues with low B-factors are particularly highlighted. On one hand, residues presenting large contributions to the conformational change will be stand out. These residues experience large structural distortions upon ligand-binding without presenting significant flexibility or uncertainties in their coordinates in the original conformational ensemble of the ligand-free native state of the protein. They are dragged by the large-scale domain movements that are triggered when the equilibrium populations of the conformational ensemble shift towards the ligand-bound state. On the other hand, residues that barely move between the ligand-free and ligand-bound conformations will be also stand out. These residues are localized in well-defined hinge regions without connecting secondary structure elements(SSE) or domains in a sequential manner, like loops, but rather participating as pivots through inter-SSE or inter-domain contacts. We expect that mutations introduced in these latter kind of residues should strongly affect the vibrational motions involved in the unbound-to-bound conformational changes. In order to confirm that we analyze the incidence of inter-SSE contacts defined as

$$I_{SSE-\;X}=\frac{\nu_{inter-SSE-X-Y}^{\text{key}}}{\nu_{inter-SSE-X-Y}}$$ (7)

where $\nu_{inter-SSE-X-Y}^{\text{key}}$ is the frequency of key positions participating in inter-SSE contacts between X and Y among those localized on X, being X = E, and H, and Y = E,B, H, G, S,T, N, and I, and *v*_inter−SSE−X−Y_ the corresponding frequency in the rest of the residues. Table 3 displays these values. We observe a large incidence of inter-SSE contacts in key positions, confirming our hypothesis that these residues participate of inter-SSE contacts between well-structured strands and helices.

**Table 3:** Incidence of different residues in key positions participating in inter-SSE contacts.

E-H	2.83096	H-B	2.70408
E-N	1.92404	H-E	2.19460
E-E	1.89671	H-H	1.62143
E-B	1.88451	H-S	0.86975
E-S	1.71122	H-T	0.83943
E-G	1.56960	H-G	0.81984
E-T	1.03616	H-N	0.76340
E-I	0.00000	H-I	0.00000

Our present analysis does not depend on neither protein sequence information nor on the analysis of evolutionary conservation and structural-mapping of phylogenetic information as evolutionary trace methods. We do not attempt to compete with previous methods developed for the prediction of ligand-binding sites[73,74]. The functionality of our key position residues is not necessarily related to direct protein-ligand interactions or catalytic activity but the conformational diversity associated to ligand-binding. Therefore, it is not expected that all mutations presenting effects on either the affinity for substrate and catalytic activity can be associated to our definition of key position residues that involves residues associated to a very particular aspect of the protein functionality, that is, vibrations associated to structural distortions introduced by ligand-binding. In order to analyze that, we have compared our results with experimental data from information provided by UniProt database [75]. UniProt provides a complete overview of the information available about proteins including information related to function, catalytic activity, and mutations with reported effects on either the affinity for substrate and catalytic activity. Uniprot contains information about 185 mutations for 43 proteins of our dataset. Only 13 of these mutations in 11 proteins correspond to key position residues. This result is something expected since, as we have previously reported, only ∼10% of the key position residues correspond to active site residues. That is, our predicted key residues do not match with catalytic residues. Considering that our procedure allows the identification of key spatial regions rather than individual residues, we have extended our analysis in order to include residues that are in direct contact with key position residues according to RING[67]. In that way, we found that 98 of the Uniprot reported mutations are in agree with our findings. That is 53% of mutations with any kind of experimental evidence related to ligand-affinity and enzyme catalysis match, or are in close contact with, key position residues that sustain the conformational diversity associated to ligand binding.

**Fig 6:**
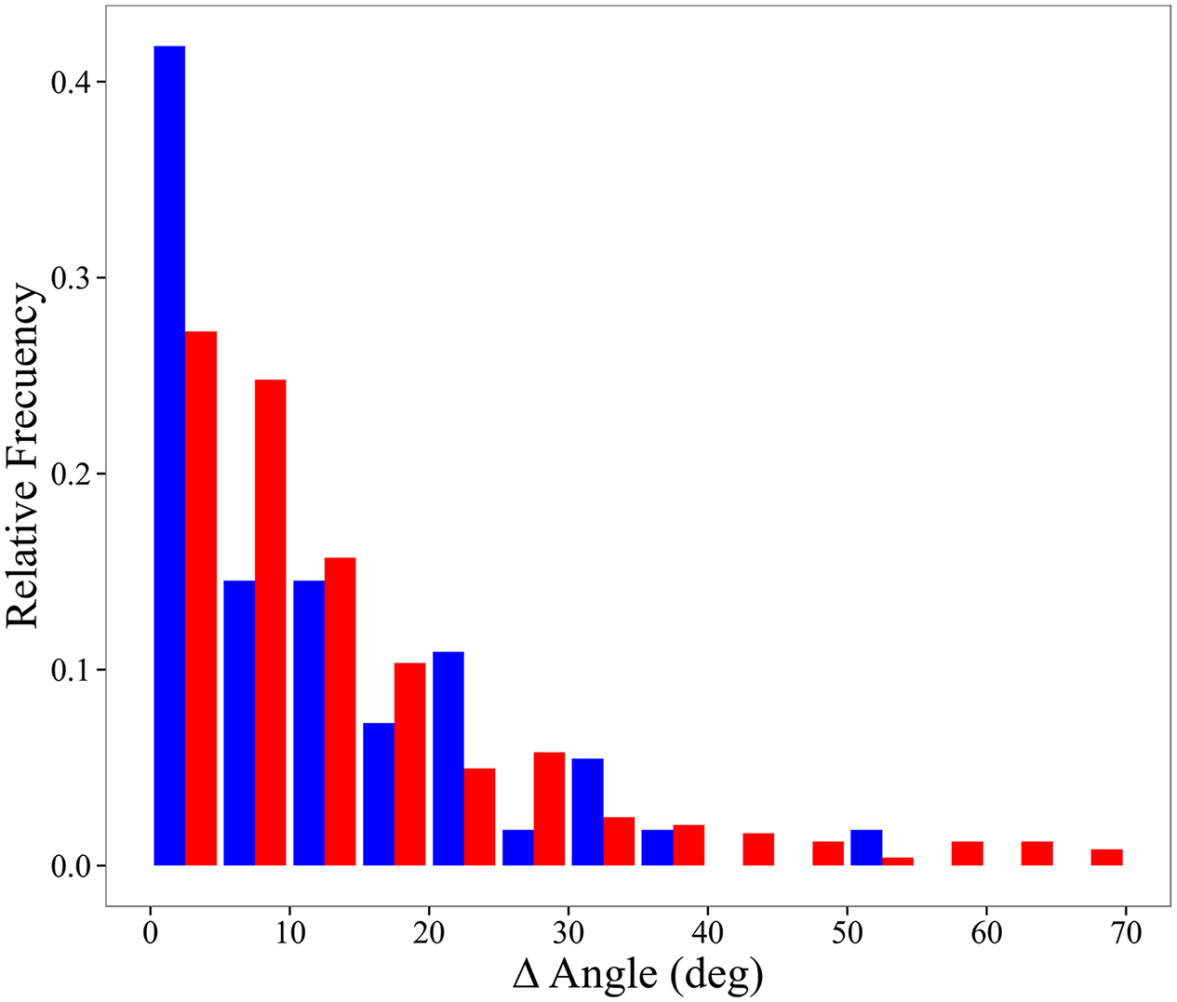
Distribution of the largest difference among the angles formed by the corresponding inertial axis of individual SSEs connected through a key position (red), and through other residues (blue).

In order to further analyze the role of key positions as pivots between SSEs we used a similar approach to that previously used to investigate domain movements between ligand-free and ligand-bound conformers[76]. Considering a key residue belonging to a SSE X and performing an inter-SSE contact with a SSE Y, we calculate the difference between angles formed by the corresponding inertial axis of individual X and Y in ligand-free and ligand-bound structures. We choose the largest difference among them as a quantitative measure of differences of SSE relative orientation. More details can be found elsewhere[76,77]. Our results, shown in Fig 6, indicates that SSEs that are connected through a key position present larger angular movements compare to those in which no key position participates in the inter-SSE contact.

It is interesting to note that Fig 6 relates key position residues with observed structural distortions introduced by ligand binding. Differences in the angular motions are directly obtained from the PDB coordinates of the ligand-free and ligand-bound structures. Therefore, the use of a simplified coarse-grained potential, based on a description of the protein as an elastic network of α-carbons, do not bias these relative displacements between SSEs.

**Fig 7:**
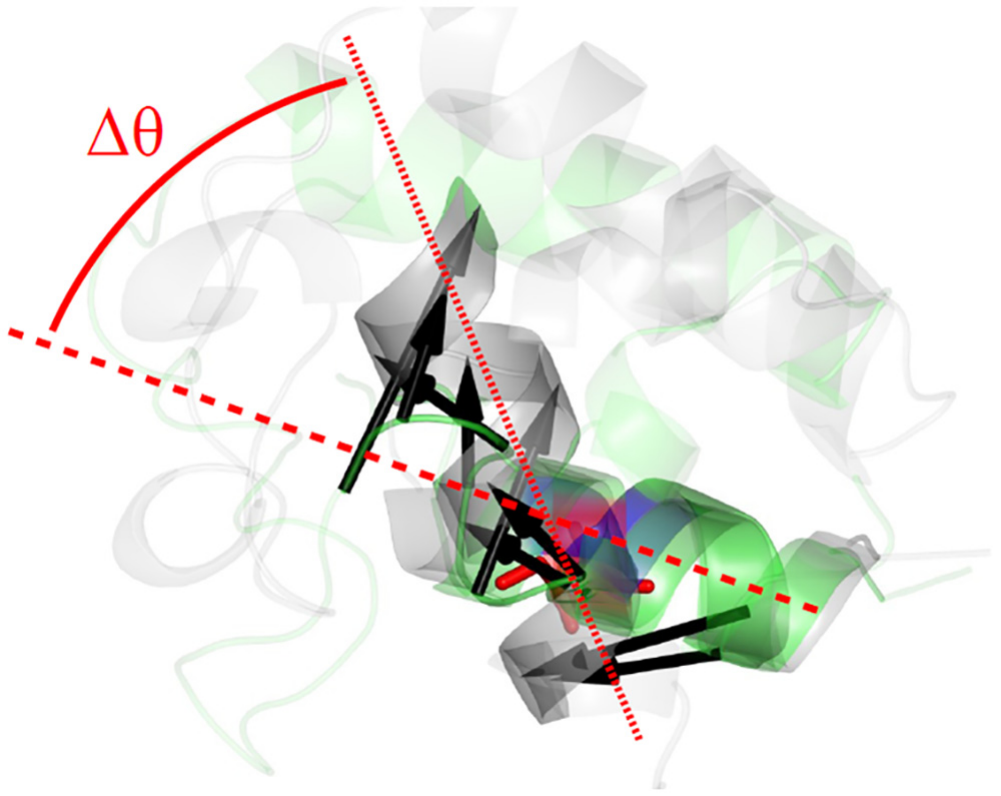
Change in the relative orientation between two α-helices in *Escherichia coli* acyl carrier protein (ACP). Ligand free (PDBid: 1ACP, chain A) and ligand-bound (PDBid: 2FAE, chain B) are depicted in green and gray respectively. The key position residue I69(red) participates of an H-H inter-SSE contact with V7(blue). The arrows indicate the directions in which residues move during the conformational transition upon ligand binding. Δθ = θ—θ’, being θ and θ’ the angles between H4 α-helix (Q66-H75) and H1 α-helix (E4-Q14) in ligand free and ligand-bound structures respectively.

In order to clarify the role that inter-SSE contacts mediated by key position residues have on the conformational transition upon ligand binding, Fig 7 shows the case of the *Escherichia coli* acyl carrier protein (ACP) as an example of a key position participating of an H-H inter-SSE contact. This ACP is a 77 amino acid protein involved in fatty acid synthesis (PDB codes 1ACP and 2FAE for ligand-free and ligand bound structures, respectively [78, 79]). Fig 7 shows key position residue I69 localized in H4 α-helix (Q66-H75). Residue I69 interacts with V7 belonged to H1 α-helix (E4-Q14). The arrows indicate the directions in which residues move during the conformational transition upon ligand binding. The angle Δθ indicates the change in the relative orientation between H1 and H4, with I69 participating as pivot through inter-SSE contact with V7.

### C. Examples

**Fig 8:**
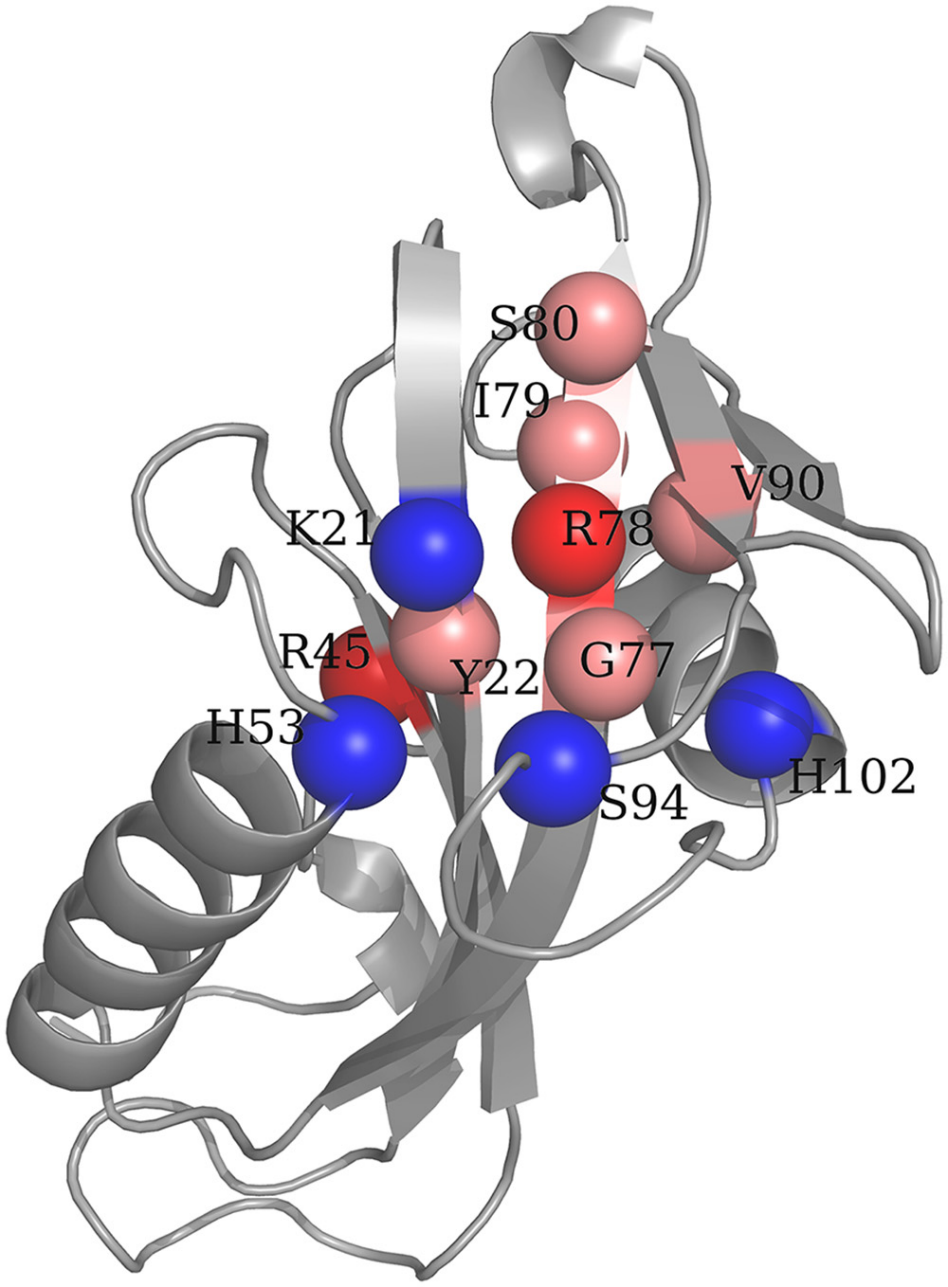
Ligand-free structure of PHPT1. Residues are colored as follows: Key position residues (pink), residues identified by UniProt[75] whose mutations affect the affinity for substrate and catalytic activity (blue), and key position residues identified also by Uniprot (red).

To provide a view of our findings, a coupled of selected cases are discussed. The first example is the human protein histidine phosphatase 1 (human PHPT1) (PDBid: 2AI6 and 2OZWf for ligand-free and ligand bound structures, respectively [80]. This 125 amino acid enzyme plays important roles in signal transduction and other cellular functions. Fig 8 displays PHPT1 structure in its apo form. The active site is located between helix α1 and loop L5.

Seven evolutionary conserved key position residues have been identified as dynamically important sites that mediate the ligand-binding conformational change: Y22, R45, G77, R78, I79, S80, V90. According to information provided by UniProt database [75], mutations on K21, R45, H53, R78, S94, and H102 have effects on either the affinity for substrate and catalytic activity. In Fig 8 key position residues and residues identified by UniProt are indicated. As can be seen, most of key position residues correspond to, or are in contact with, residues whose mutations are experimentally confirmed to alter the affinity for substrate and catalytic activity.

**Fig 9:**
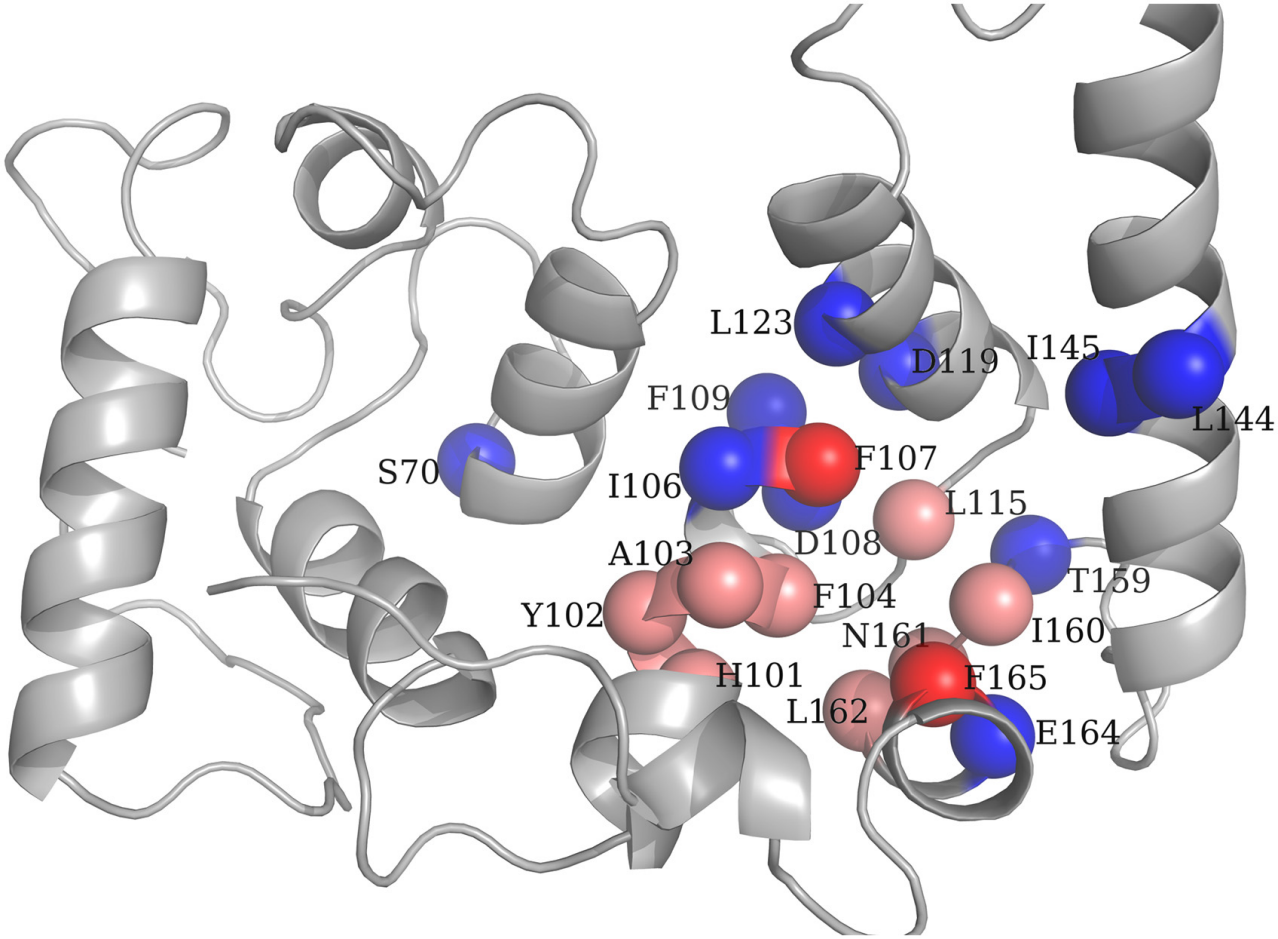
Ligand-free structure of CIB1. Residues are colored as follows: Key position residues (pink), residues identified by UniProt[75] whose mutations affect the affinity for substrate and catalytic activity (blue), and key position residues identified also by Uniprot (red).

A second example that illustrates our findings corresponds to the calcium- and integrin-binding protein 1 (CIB1) (PDBid: 1DGU and 1Y1A for ligand-free and ligand bound structures, respectively [81,82]. This enzyme has 183 residues. CIB1 binds to the 20-residue α_IIb_ cytoplasmatic domain of platelet α_IIb_β_3_ integrin. It acts as a global signaling regulator on a wide variety of proteins in cells in addition to platelets. Fig 9 shows CIB1 structure in its apo form.

Ten evolutionary conserved key position residues have been selected: H101, Y102, A103, F104, F107, L115, I160, N161, L162, F165. As it has been previously pointed out, our procedure allows the identification of spatial regions H101-F107 and I160-F165 rather than individual residues. Positions that present experimental evidence of mutations that impact on ligand-binding and catalytic activity are: S78, I106-F109, D119, L123, L144, I145, T159, E164, and F165. All these residues are indicated in Fig 9 It is important to stress that effects on the affinity for substrate and catalytic activity are not necessarily associated to effects on the conformational diversity of the protein. Our key position residues are associated to a very particular aspect of the protein functionality, that is, vibrations associated to structural distortions introduced by ligand-binding. Despite that, both key spatial regions H101-F107 and I160-F165 are validated by experimental evidence.

Finally, the effect of mutations on key position residues has been analyzed using the recently developed Elastic Network Contact Model (ENCoM) [83] that employs a potential energy function that includes a pairwise atom-type non-bonded interaction term. In both cases, human PHPT1 and CIB1, the predicted variations in free energy variations (ΔΔG), evaluated with ENCoM and FoldX [84] indicate that mutations on key position residues correspond to destabilizing mutations, that is, mutations that affect stability due to a decrease in the entropy of the folded state. The average ΔΔG considering all possible mutations on each key position residues were 2.0 kcal/mol and 1.3 kcal/mol for human PHPT1 and CIB1 respectively. Selecting the most destabilizing mutations ΔΔG_max_ on each key position residues, we obtained an average of 4.8 kcal/mol and 3.6 kcal/mol for human PHPT1 and CIB1 respectively. That is, in both cases, key position residues involve residues whose mutations can drastically affect the protein structure.

## Methods

### A. Protein’s dataset

We obtained pairs of conformers in their bound and unbound form from the database of Conformational Diversity in the Native State of proteins (CoDNaS)[34]. This database is a collection of redundant structures for the same protein, obtained from different experimental protocols. CoDNas is linked with physicochemical and biological information allowing to explore how different parameters modulate protein conformational diversity. The maximum C-alpha root-mean-square-deviation (RMSD) value is considered as a measure of the conformational diversity extension. In the present work, we have retrieved pairs of structures of the same protein whose unique difference in the structure estimation is the presence or absence of ligand. Each pair of ligand-free and ligand-bound structures corresponds to the pair with maximum structural difference among all possible pairs according to their C-alpha RMSD.

**Fig 10:**
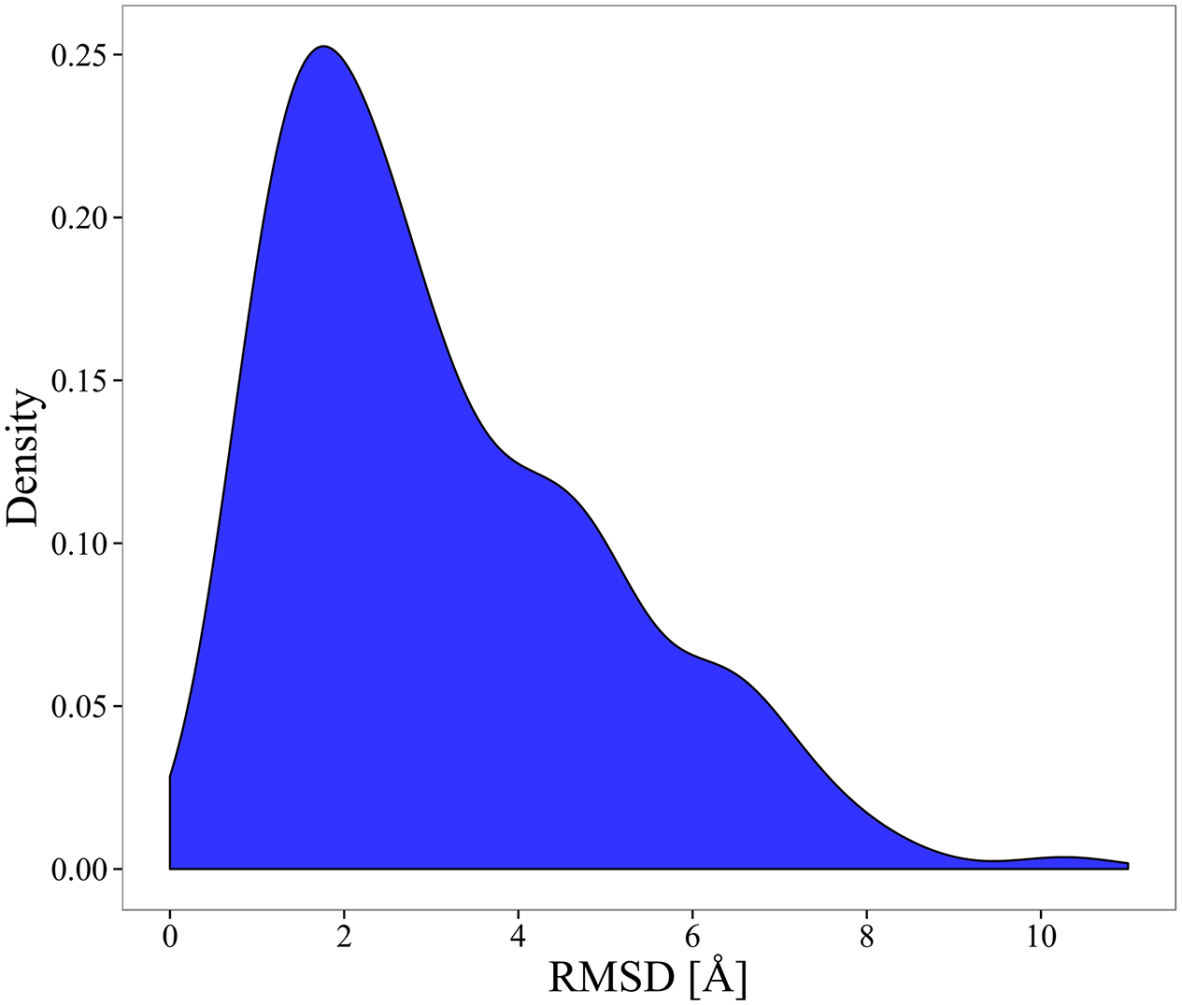
Distribution of the RMSD values over all pairs of ligand-free and ligand-bound conformations.

We applied several filters in the original dataset in order to obtain a well curated dataset: (i) crystal structures with resolution < 4 Å, (ii) structures without missing residues in the pdb files, (iii) crystal structures with optimal Spearman rank correlation coefficient between experimental and theoretical B-factors > 0.4 Å, (iv) proteins whose coverage in the multiple alignment obtained using HSSP[85] database of protein structure-sequence alignment is ≥ 80%, (v) proteins with more than 100 homologous in the HSSP alignment. Therefore, finally we obtained a total of 188 pairs of ligand-free and ligand bound protein structures. Fig 10 displays the distribution of the RMSD values obtained over all pairs of the final selected dataset. The list of the pairs with their corresponding PDB code is provided in S1 Table.

### B. Elastic Network Models background

The Elastic Network Models (ENM) describe the protein as an elastic network of α-carbons linked by springs within a cutoff distance *r*_*c*_. Here in, the value of *r*_*c*_ is varied from 7Å to 20Å in order to optimize the correlation between theoretical and experimental B-factors.

The locations of the α-carbons in the crystallographic structure are considered as the equilibrium positions, about which the atoms fluctuate. The interaction between residues are described by the simplified coarse-grained potential[36, 59,86]

$$E\left(r_{i},r_{j}\right)=\frac{1}{2}k_{ij}{\left(\left|r_{ij}\right|-\left|r_{ij}^{0}\right|\right)}^{2}$$ (8)

with ***r***_*ij*_≡ ***r***_*i*_−***r***_*j*_ being the vector connecting atom *i* and *j*, and the zero superscript indicates the equilibrium position. In order to take account of the chemical interactions, the value of the force constant *k*_*ij*_ is determined according to the following rules[87]:

if |*i*−*j*| = 1 ⇒ *k*_*ij*_ = *γ*

else

if $\left|r_{ij}^{0}\right|\leq r_{c}$ then

 if *i* and *j* are connected by disulphide bridge ⇒ *k*_*ij*_ = *γ*

 if *i* and *j* interact by hydrogen bond or salt bridge ⇒ *k*_*ij*_ = *γ* x 0.1

 otherwise ⇒ *k*_*ij*_ = *γ* x 0.01

if $\left|r_{ij}^{0}\right|\geq r_{c}$ ⇒ *k*_*ij*_ = 0

being *γ* a scaling constant to match the theoretical result to experimental data. We use CSU program[88] to obtain the connectivity information related to hydrogen bonds, salt bridges, and disulphide bridges.

The potential energy of a protein with *N* residues can be expressed as a *N*x*N* matrix **E** with elements *E*(***r***_*i*_,***r***_*j*_). Normal modes are obtained by diagonalizing the second-order partial derivatives or Hessian matrix **H** of **E** as

$$\Lambda =\;q^{T}Hq$$ (9)

where **q** is an orthogonal *N*x*N* matrix whose columns **q**_k_ are the eigenvectors of **H**, that is, the normal modes, and **Λ** is the diagonal matrix of eigenvalues λ_k_ of **H**. The temperature factor or B-factor *B*_*i*_ of atom *i* is proportional to the mean square displacement $\langle \Delta r_{i}^{2}\rangle =\langle {\left(r_{i}-r_{i}^{0}\right)}^{2}\rangle$ from its equilibrium position[89]

$$B_{i}=\frac{8\pi^{2}}{3}\langle \Delta r_{i}^{2}\rangle$$ (10)

and it can be expressed as the sum of contributions from the 3N-6 internal modes of motion {**q**_*k*_}_k = 1,3N−6_ as[90]

$$\langle \Delta r_{i}^{2}\rangle =3k_{B}T\Sigma_{k=1}^{3N-6}{\left[\lambda_{k}^{-1}q_{k}q_{k}^{T}\right]}_{ii}$$ (11)

where *k*_*B*_ is the Boltzmann constant, *T* is the absolute temperature.

### C. Normal mode subspaces associated to ligand-binding

Normal modes most involved in the conformational change are selected according to their corresponding overlap with structural distortions introduced by ligand binding. In this section, we describe the procedure we follow in order to define the subspace composed by these modes.

Firstly, the pair of ligand-free and ligand-bound structures is superimposed minimizing the RMSD. The normalized difference vector **v** between these reoriented structures retains the direction of the observed structural change upon ligand binding. Nevertheless, many proteins contain unstructured or flexible regions such as loops whose coordinates are not well experimentally resolved. Actually, amino and carboxyl ends of proteins are particularly flexible, but this flexibility is not associated with biological causes. In order to reduce the possibility that our results can be skewed by any structural distortion not directly related to ligand binding, we use a Gaussian-weighing factor[91] in the construction of **v** whose elements are defined as

$$v_{i}=\frac{\left(y_{i}-x_{i}\right)e^{-\frac{\left(B_{i}^{\text{lf}}+B_{i}^{\text{lb}}\right)}{w}}}{{\sum^{\text{​}}}_{j}^{3N}{\left(\left(y_{i}-x_{i}\right)e^{-\frac{\left(B_{i}^{\text{lf}}+B_{i}^{\text{lb}}\right)}{w}}\right)}^{2}}$$ (12)

where the ligand-free and ligand-bound conformations are represented by C_α_ coordinate sets {*x*_*i*_} and {*y*_*i*_} respectively, *N* is the total number of residues of the protein, $B_{i}^{\text{lf}}$ and $B_{i}^{\text{lb}}$ are theoretical B-factors in the ligand-free and ligand-bound conformations respectively, and *w* is an arbitrary scaling factor.

Next, the normalized difference vector **v** is expanded on the basis of ligand-free normal modes

$$v=\Sigma_{k=1}^{3N-6}\left(v\cdot q_{k}\right)q_{k}=\Sigma_{k=1}^{3N-6}\left(\Sigma_{j=1}^{3N}\left(v_{j}q_{jk}\right)\right)q_{k}=\Sigma_{k=1}^{3N-6}c_{k}q_{k}$$ (13)

with

$$c_{k}=\Sigma_{j=1}^{3N}\left(v_{j}q_{jk}\right)$$ (14)

The degree of delocalization of **v** among the different ligand-free normal modes can be obtained evaluating the mode participation number[92,93] as

$$P_{q}={\left(\Sigma_{k=1}^{3N-6}{\left(c_{k}\right)}^{4}\right)}^{-1}$$ (15)

The participation number has been originally introduced as a convenient means of describing a measure of the delocalization for a given normal mode vector. In that case, the participation number has the value of 3*N* for a pure translation, and the value of unity for a highly localized mode. Beyond these two extremes, the participation number can be used to define the delocalization at intermediate situations. That is, the participation number represents a measure of the delocalization of the normal mode vector on the basis of the atomic Cartesian coordinates. In the present work, we extend this concept in order to apply it to the delocalization of the difference vector **v**, that takes account of structural distortions introduced by ligand binding, on the basis of ligand-free normal modes. The value of *P*_**q**_, rounded to the nearest higher integer, contains information about the number of modes needed to describe the direction of the conformational change. Values of *P*_**q**_ ≈ 3*N*−6 mean that the conformational change is spread among all vibrations of the ligand-free conformer, that is, the full space of normal modes is required in order to achieve a good representation of the conformational change. Values of *P*_**q**_ ≈ 1 indicate that one single normal mode dominates the direction of the conformational change. The first *P*_**q**_ modes ordered by index *f*_*k*_ in decreasing values of (*c*_*k*_)^2^ define the minimum subspace **S** of modes ${\left\{q_{f_{i}}\right\}}_{i=1,Pq}$ required to achieve a good description of the conformational change. In this way, **S** retains normal modes most involved in the ligand-binding conformational change. That is, size and composition of subspaces **S** associated to the conformational change are defined by *P*_**q**_ and the set of *P*_**q**_ ligand-free normal modes that contributes the most to the unbound-to-bound conformational change, respectively.

### D. Local perturbations

The effect of point mutations of a residue *i* on the subspace **S** of ligand-free normal modes associated to ligand-binding is simulated by introducing perturbations to the local interactions involving the *i*^th^ residue. Following the procedure previously applied in the Structural Perturbation Method (SPM) by W. Zheng et al.[50,55,57, 94], the force constants *k*_*ij*_ that connect *i* with other residues *j* are changed by a small amount δγ. Then, a new set of normal modes ${\left\{q_{k}^{i}\right\}}_{k=1,3N-6}$ is obtained.

In order to define the new subspace **S**^**i**^ it is necessary to establish a one-to-one correspondence between both unperturbed and perturbed set of modes. Perturbations to the local elastic interactions can lead to changes in the energy order of the modes. Because of that, the assignment of the perturbed modes based on the energy-ordering criterion becomes useless. The correspondence between both sets of modes, $\left\{q_{k}^{i}\right\}$ and {**q**_*k*_}, can be based on the highest values of their overlaps. The maximum overlaps are obtained through the maximization of the trace of the square of the overlap matrix **O** whose elements are defined as the dot product

$${\text{O}}_{kk^{\prime }}=q_{k}^{T}\cdot q_{k^{\prime }}^{i}$$ (16)

This can be done by selecting those elements of the **O** matrix, one for each row, and each pertaining to a different column (or vice versa), which maximize the sum of their squared values. In order to do that, we have used a variant of the Min-Cost algorithm[58,95].

### E. Comparison of normal mode subspaces

The comparison of unperturbed and perturbed subspaces of modes, **S** and **S**^**i**^ (see Section C and D), associated to the conformational change upon ligand-binding can be performed through the calculation of the corresponding Gramian matrix[96,97, 98,99] as follows. We define the matrices **S**(3*N x M*) and **S**^**i**^ (3*N x M*) associated to the unperturbed and perturbed subspaces with vector columns of *M* modes {**q**_*k*_}_k = 1,M_ and ${\left\{q_{k}^{i}\right\}}_{k=1,M}$ containing the set of *M* modes selected according to the procedures described previously in Section C and D. These matrices can be compared by defining the vector projection of each $q_{j}^{i}$ onto the set of modes {**q**_*k*_}_k = 1,M_ as

$$p_{j}^{S^{i}S}=\Sigma_{k=1}^{M}\left(q_{j}^{i}\cdot q_{k}\right)q_{k}$$ (17)

The Gramian matrix **G** (*M x M*) of the set of vectors ${\left\{p_{j}^{S^{i}S}\right\}}_{j=1,M}$ is calculated as the matrix of inner products with elements

$${\text{G}}_{kl}=\left(p_{k}^{S^{i}S}\cdot p_{l}^{S^{i}S}\right)$$ (18)

The diagonalization of **G**

$$L_{G}^{T}GL_{G}=\Lambda_{G}$$ (19)

allows us to use the eigenvalues of **G**, {λ_*k*_}_*k = 1*,*M*_, as a measure of the similarity between the two subspaces. Since all the eigenvalues of **G** varies between 0 and 1[96], we can define a measure of the similarity of the two subspaces as

$$\zeta^{S^{i}S}=\frac{{\sum^{\text{​}}}_{k}^{M}\lambda_{k}}{M}$$ (20)

The smaller the value of $\zeta^{S^{i}S}$, the stronger the effect that mutations in the *i*^th^ residue will have on the subspace of modes associated to the conformational change upon ligand-binding, that is, the required conformational diversity of the protein will be less guaranteed. The value of $\zeta^{S^{i}S}$ increases with the dimensionality of the subspace **S**. To solve this problem, for each protein in the dataset we normalize the values of $\zeta^{S^{i}S}$ as:

$$Z_{\text{score}}^{S^{i}}=\frac{\zeta^{S^{i}S}-\bar{\zeta^{S^{i}S}}}{\sigma^{S}}$$ (21)

where $\bar{\zeta^{S^{i}S}}$ and σ^**s**^ are the average and standard deviation of the distribution of $\zeta^{S^{i}S}$ over all residues.

### F. Key position residues

Key positions are selected as those ranked with the lowest 5% values of $Z_{\text{score}}^{S^{i}}$ for each protein in the dataset. Other choices for this cut off value between 1% and 10% have also been tested without obtaining qualitatively differences in our results. In this way, the set of key positions per pair of ligand-free and ligand-bound conformers is associated to directions of conformational changes rather than absolute values of observed structural distortions. The number of key position residues per pair of conformers in our dataset is given in S1 Table.

### G. Protein sequence-structure alignments

Multiple structure-sequence alignments were obtained from the HSSP (homology-derived structures of proteins) database[85] that merge structural and sequence information of proteins. We have only selected sequences with a coverage greater than 80%. The analysis of conservation of each aligned position has been performed using Henikoff entropy measure[100,101] to estimate position-specific amino acid frequencies. The resulted conservation index for each position are normalized obtaining the corresponding z-score value, $Z_{\text{score}}^{\text{evol},\text{i}}$, as the final parameter related to the evolutionary conservation of the *i*^th^ residue of the protein.

### H. Characterization of residues

Relative solvent accessibility (RSA) values are calculated using the NACCESS program[66]. A residue is considered exposed if its relative accessibility is ≥10%. The relative accessibility is computed as the percent of the computed accessibility of a residue out of the accessibility of that amino acid in an extended ALA-X-ALA tripeptide (where X is the type of amino acid)[102,103].

The number of inter-residue contacts for each residue of the dataset are calculated using RING[67]. This is a web tool for analysis of protein structures in terms of physico-chemical interactions. For each protein we generate an all interaction networks, with a cutoff distance of 5 Å.

Finally, BioLip database[68] has been used to obtain information concerning ligand binding site of each protein in the dataset. For the calculation of distance to ligand binding site, we first identify the presence of more than one binding site and we generate a center of mass from the coordinates of all the amino acids that make up the binding site. Second, we determine the distance of each residue (α-carbon) to the centre of mass for each binding site and the minimum distance is selected.

### Conclusions

Conformational diversity of the native state of a protein involves a dynamical equilibrium between conformers with lower (ligand-free) and higher (ligand-bound) affinities for the ligand. Internal protein motions guarantee the interconversion between them. Due to its relevance to protein function, conformational diversity associated to ligand binding should be evolutionary conserved. Here, we have presented a novel procedure to identify key positions whose mutations have a significant effect on vibrational normal modes involved in the ligand-free to ligand-bound conformational changes. We have applied our method to a refined dataset of paired protein structures in the ligand-free and ligand-bound form.

In order to avoid normal mode mixtures and/or rearrangements in their frequency ordering introduced during ligand-binding, we deal not with individual normal modes but with normal mode subspaces associated to ligand-binding. We have described a procedure to define and compare these subspaces. Furthermore, our definition of key positions, i.e. positions that are dynamically important to ligand-binding, is based on the effect of mutations on these subspaces.

We find a negative correlation between the effects of site-specific mutations on the subspaces of normal modes associated to ligand-binding and the evolutionary conservation of these sites. Residues whose mutations are found to alter the most these subspaces are defined as key positions, that is, dynamically important positions that mediate the ligand-binding conformational change. We also found that they correspond to buried aliphatic residues mostly localized in regular structured regions of the protein like β-sheets and α-helix. Furthermore, they seem to participate as pivots through inter-SSE contacts.

Key position residues are identified using subspaces of collective vibrations that participate in a specific conformational change. These collective vibrations are commonly low-frequency normal modes involving the concerted motion of residues that can be localized in well separated spatial regions of the protein structure. Therefore, the method is not affected by any bias that can overestimate the effect of residues localized close to the binding-site. Because of that, we have shown that only ∼10% of the key position residues correspond to active site residues. That is, active-site residues only comprise a small fraction of the predicted key residues.

Our key position residues are associated to a very particular aspect of the protein functionality, that is, vibrations associated to structural distortions introduced by ligand-binding. In that sense, the analysis provides distinct and complementary information respect to studies based on the identification of sequential and structural active site similarities among homologous proteins.

Furthermore, the method is not restricted to identify key position residues whose mutations directly affect the affinity for substrate. It can be straightforward applied to identify key position residues whose mutations affect oligomerization binding constants and stability, inter-protein interactions, and allosteric responses among others. Further applications of the method to these other aspects of protein function are in progress.

As protein function resides in conformational transitions, we think that our method to estimate key positions related with protein dynamics, could help us to improve our understanding on structure-function relationship as well as functional diversification during evolution.

## Supporting Information

S1 Table Protein’s dataset. (XLSX)
